# Laser-induced elastic wave classification: thermoelastic versus ablative regimes for all-optical elastography applications

**DOI:** 10.1117/1.JBO.25.3.035004

**Published:** 2020-03-18

**Authors:** Susobhan Das, Alexander Schill, Chih-Hao Liu, Salavat Aglyamov, Kirill V. Larin

**Affiliations:** a University of Houston, Department of Biomedical Engineering, Houston, Texas, United States; b University of Houston, Department of Mechanical Engineering, Houston, Texas, United States

**Keywords:** optical coherence elastography, thermoelastic, ablative, all-optical, elastic waves

## Abstract

**Significance**: Shear wave optical coherence elastography is an emerging technique for characterizing tissue biomechanics that relies on the generation of elastic waves to obtain the mechanical contrast. Various techniques, such as contact, acoustic, and pneumatic methods, have been used to induce elastic waves. However, the lack of higher-frequency components within the elastic wave restricts their use in thin samples. The methods also require moving parts and/or tubing, which therefore limits the extent to which they can be miniaturized.

**Aim**: To overcome these limitations, we propose an all-optical approach using photothermal excitation. Depending on the absorption coefficient of the sample and the laser pulse energy, elastic waves are generated either through a thermoelastic or an ablative process. Our study aimed to experimentally determine the boundary between the thermoelastic and the ablative regimes for safe all-optical elastography applications.

**Approach**: Tissue-mimicking graphite-doped phantoms and chicken liver samples were used to investigate the boundary between thermoelastic and ablative regimes. A pulsed laser at 532 nm was used to induce elastic waves in the samples. Laser-induced elastic waves were detected using a line field low coherence holography instrument. The shape of the elastic wave amplitude was analyzed and used to determine the transition point between thermoelastic and ablative regimes.

**Results**: The transition from the thermoelastic to the ablative regime is accompanied by the nonlinear increase in surface wave amplitude as well as the transformation of the wave shape. Correlation between the absorption coefficient and the transition point energy was experimentally determined using graphite-doped phantoms and applied to biological samples *ex vivo*.

**Conclusions**: Our study described a methodology for determining the boundary region between thermoelastic and ablative regimes of elastic wave generation. These can be used for the development of a safe method for completely noncontact, all-optical microscale assessment of tissue biomechanics using laser-induced elastic waves.

## 1 Introduction

Recent advancements in optical coherence elastography (OCE) technique^1^^–^^4^ have followed the progress in optical coherence tomography (OCT),^5^ with increasing performance in both imaging resolution and speed.^6^^–^^9^ Change in the phase of the complex OCT signal can be used to evaluate axial tissue displacements in both static and dynamic OCE approaches.^2^^,^^9^^–^^11^ Static, also known as compression OCE, usually relies on slow tissue deformation using contact loading methods and measuring quasistatic strain and displacement fields.^10^^,^^11^ Static approaches are capable of mapping the local two-dimensional and three-dimensional strain distribution with high spatial resolution, including estimation of the nonlinear elastic properties.^10^^–^^18^ In dynamic elastography, the tissue is perturbed by impulsive loading or harmonic vibrations, and the elasticity estimation is based on tissue motion as a function of time, i.e., velocity of the elastic wave or displacement dynamics.^2^^–^^4^ Dynamic approaches permit direct quantification of the absolute values of the elastic constants using appropriate models. Thus, dynamic elastography is gaining popularity and currently used for many medical applications.

In dynamic OCE, however, the mechanical excitation of tissue induced by various techniques, such as direct contact,^19^ acoustic radiation force,^20^^–^^22^ or pneumatic,^23^^–^^25^ has limitations. For example, contact-based methods require direct contact and, therefore, cannot be considered as noninvasive. Pneumatic air-puff excitation does not require direct contact or a coupling medium but has a relatively low-frequency response and bandwidth, which makes it more susceptible to boundary conditions.^26^^,^^27^ It also requires moving parts and tubing to deliver the air pulse, which limits the extent to which it can be miniaturized.

All-optical excitation of elastic waves has many advantages compared to other methods.^28^^–^^34^ Similar to photoacoustics, pulsed laser irradiation is converted into elastic waves by light absorption and localized heat expansion of the tissues. This transient mechanical perturbation generates compressional and shear waves that propagate through the sample. A short laser pulse excitation generates an elastic wave that contains high-frequency components and less susceptible to boundary conditions. Also, by focusing the laser beam on the sample, the excitation spot can be very small comparing all other excitation methods. The advantages of the laser-based wave generation for *in vivo* applications also include the potential for the development of endoscopic-based OCE technology.

To generate the elastic wave, the laser pulse should be efficiently absorbed and converted into heat by the tissue. Different techniques have been used to enhance the absorption of pulsed laser energy by the sample, such as the injection of nanoparticles,^35^^–^^37^ droplets of perfluorocarbon,^38^ and doping with graphite.^34^ Naturally, the endogenous mechanism of the elastic wave generation would be preferred for many biological and clinical applications.

Depending on the pulsed laser fluence and the absorption coefficient of the sample, the mechanism for generating elastic waves can be classified into two regimes: thermoelastic and ablative.^34^ While thermoelastic and ablative mechanisms for compressional wave generation in metals have been intensively investigated in the past,^39^^–^^45^ there are very few reports on generation of elastic waves in soft tissues.^34^^,^^46^ The thermally induced disturbance on a sample by low laser irradiation is mainly a result of the thermal expansion of the sample material. But, at high laser irradiation, the excitation area experiences an ablation process that causes a nonlinear dependence between laser energy and the displacement amplitude, as well as damage of the sample. Therefore, understanding the transition point between thermoelastic and ablative regimes is required for safe biological application of laser excitation in elastography. Here, we investigated the boundary between thermoelastic and ablative regimes in soft tissue-mimicking phantoms and chicken liver using a 6-ns pulsed laser at 532-nm wavelength.

## 2 Materials and Methods

**Fig. 1:**
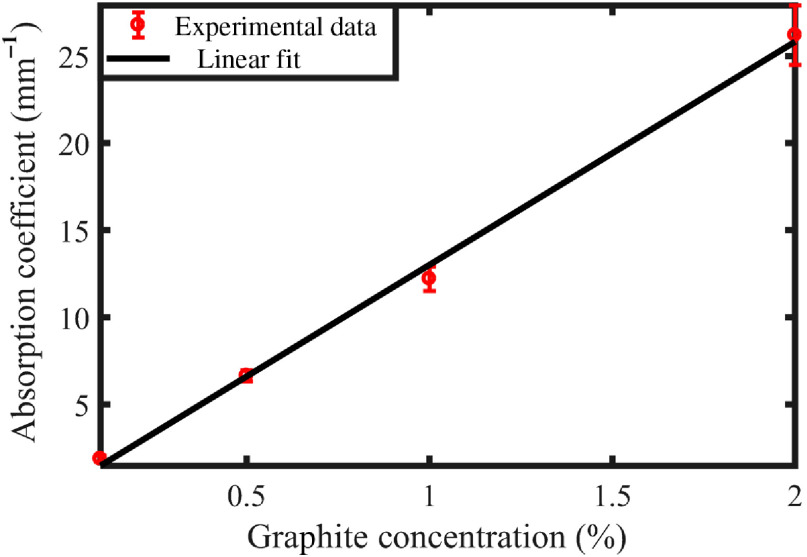
Absorption coefficient of tissue-mimicking agar phantom sample with different concentrations of graphite.

Tissue-mimicking phantoms were made of 1% (w/w) agar^23^ and prepared at different absorption coefficients by varying the percentage of graphite powder in the phantom mixture. The absorption coefficient at the pulsed laser wavelength (532 nm) of each sample was determined by measuring the light transmission of 10 slices of the phantom with different thicknesses. The mean value and the standard deviation of the measured absorption coefficient for different graphite concentrations are shown in Fig. 1 along with the linear fit.

Chicken liver from a local supermarket was used as an example of a biological sample with strong endogenous absorption at 532 nm. To avoid dehydration of the liver tissue, phosphate-buffered saline was regularly applied.

**Fig. 2:**
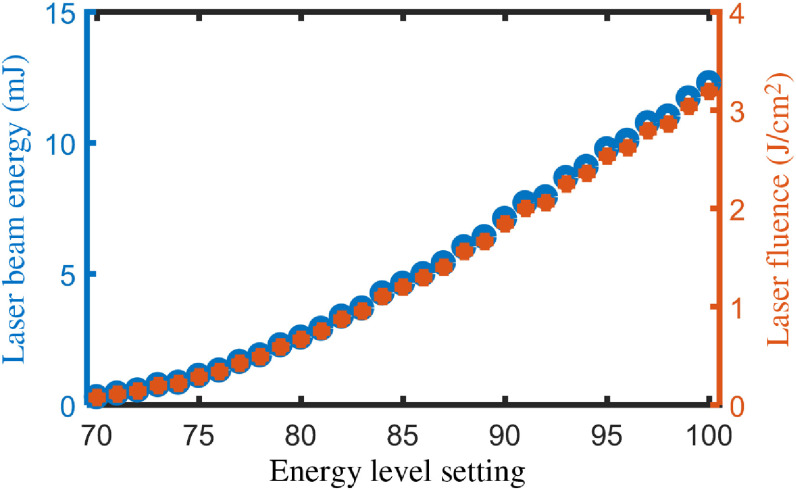
Tunable pulse laser energy and fluence on the sample for different energy level settings.

In these experiments, a Q-switched, frequency-doubled Nd:YAG laser at 532 nm (Polaris II, New Wave Research, Inc.) was used to induce elastic waves in the samples. Energy per pulse was adjusted from 0.29 to 12.27 mJ by changing the flashlamp intensity. The laser beam was focused on the sample surface with an incidence angle of ∼15  deg. The focused beam diameter on the sample surface was ∼0.7  mm. Therefore, the fluence of the pulsed laser on the sample surface was varied from 76 to $3.19{\;J/\mathrm{cm}}^{2}$. Figure 2 shows the pulsed laser energy and corresponding irradiance on the sample surface for different energy level settings of flashlamp (70 to 100 arb. un.).

**Fig. 3:**
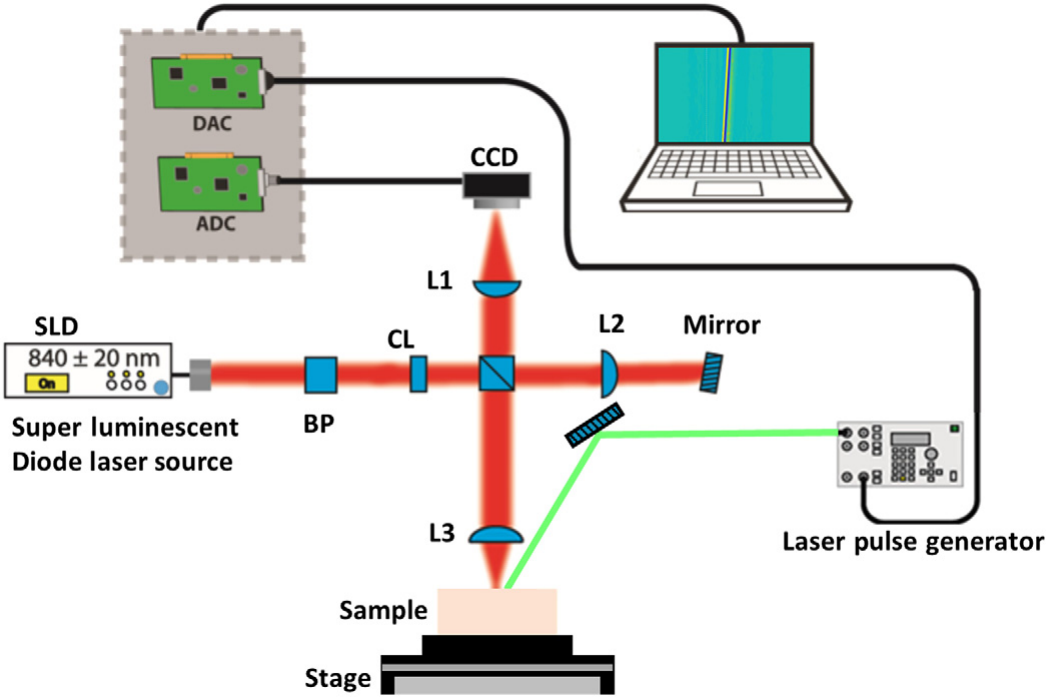
Schematic setup of LF-LCH system (top view). CL, cylindrical lens; L1 to L3, plano-convex lens; DAC, digital-to-analog converter; ADC, analog-to-digital converter; and BP, bandpass filter.

Laser-induced elastic waves were detected using a line field low coherence holography (LF-LCH) instrument, fully described in Refs. 47 and 48. Briefly, the system (Fig. 3) uses an 840±20  nm superluminescent diode (SLD, Superlum, Ireland) as the light source and a fast line scan camera (Basler Sprinter, Germany) to track propagating surface waves. LF-LCH had a temporal resolution of 5  μs and a displacement sensitivity of <1  nm. The cylindrical lens (CL) in the beam path generated a line beam and was focused on the sample with an offset distance of ∼5  mm from the excitation beam for the phantom samples and ∼0.5  mm for the liver. With the lens L1, the interference pattern was focused on CCD, which illuminates 440 pixels. The surface wave displacements were measured over the length of 1.35 mm with a spatial resolution of 9  μm for tissue-mimicking phantom samples. For the chicken liver, the displacements were measured over the interval of 0.25 mm, and the spatial resolution was increased to 2.6  μm, which was achieved by changing the lens L3. The pulsed laser was externally triggered and synchronized with the LF-LCH system.

A 30-kHz swept-source OCT imaging system with the central wavelength of 1310 nm and −3-dB bandwidth of ∼150  nm was used to measure the thickness of the tissue-mimicking graphite sample slices for absorption coefficient calculation. The axial resolution of the system was ∼11  μm. The same system was used for imaging and calculating heterogeneous absorption of the chicken liver tissue sample.

## 3 Results

**Fig. 4:**
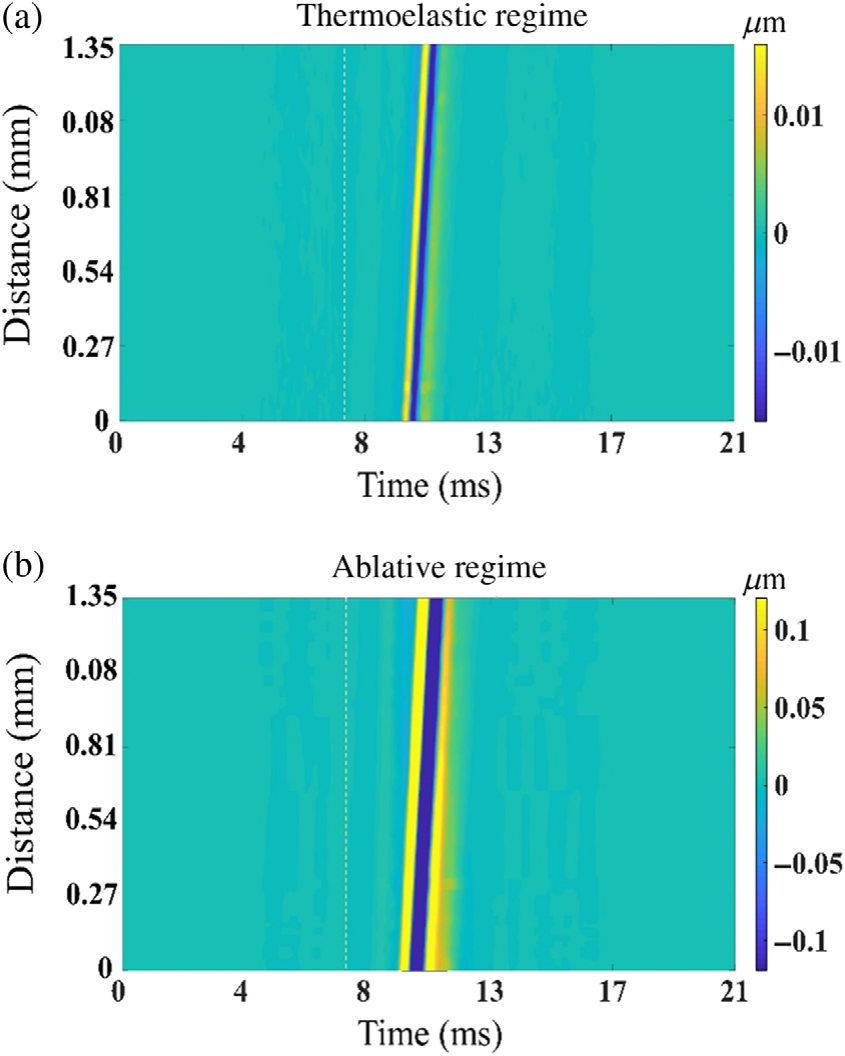
Spatiotemporal displacement profiles in (a) thermoelastic regime (1.64 mJ) and (b) ablative regime (6.01 mJ) on the surface of the 1% graphite phantom. The classification of the profiles is discussed later in the text.

The complete spatiotemporal maps of elastic waves propagating in a 1% graphite phantom in (a) thermoelastic and (b) ablative regimes are shown in Fig. 4. The dotted line in Fig. 4 indicates the time of laser excitation. There is a lag of ∼2.3  ms between the excitation pulse and the appearance of the elastic wave. The lag is proportional to the offset distance between the probe beam and the laser excitation location. These spatiotemporal maps indicate that the LF-LCH system can robustly detect the laser-induced elastic waves.

**Fig. 5:**
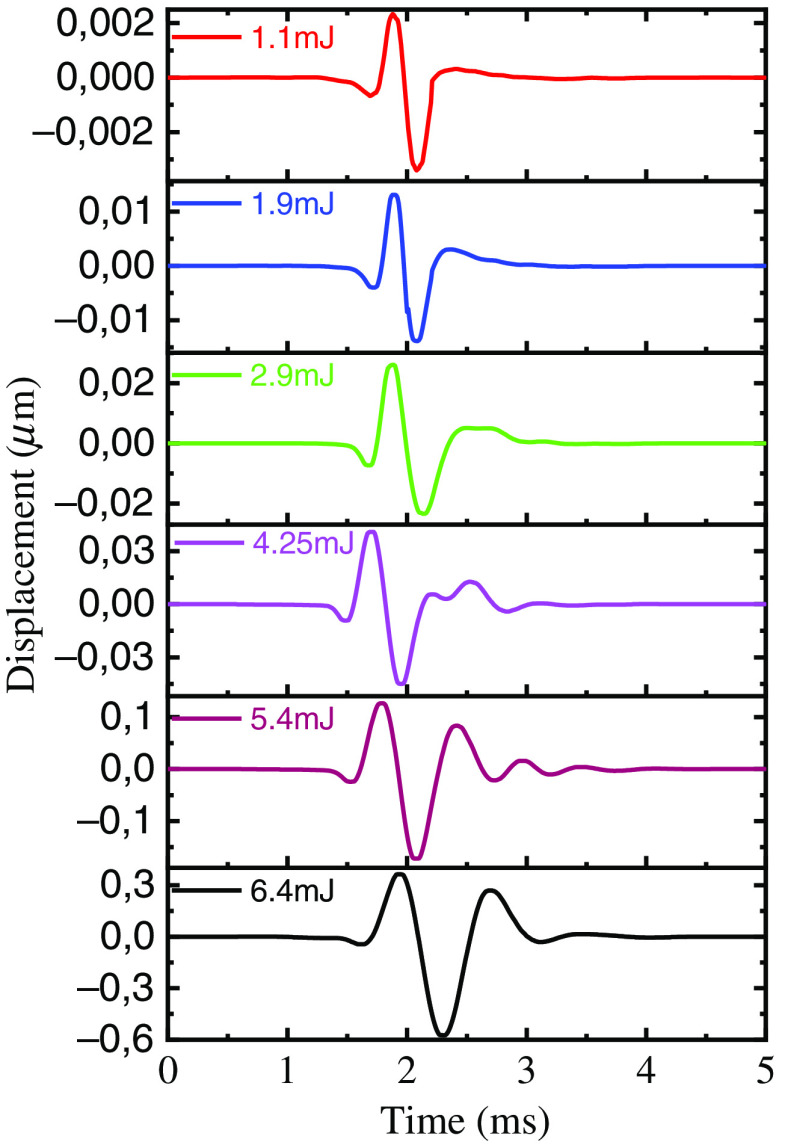
Temporal displacement profiles on the surface of 1% graphite phantom at different laser energy levels. Displacement profiles are measured at a distance of 5 mm from the laser excitation location.

Representative temporal displacement profiles of the elastic wave in 1% graphite phantom as a function of excitation energy were taken from the full spatiotemporal maps and are shown in Fig. 5. Here, a change in the temporal profile of the elastic wave is observed with increasing excitation energy. As a convention for the following discussion, outward displacement from the surface of the sample is positive (+), while inward displacement is negative (−). At lower excitation energies, positive and negative displacement features in the elastic wave profile are both: (1) equal in number (quantity) and (2) equal or nearly equal in amplitude so, therefore, become “mirror symmetric” across the zero displacement axis. As excitation energy increases, positive and negative displacement features in the elastic wave profile become both: (1) unequal in number and (2) unequal in amplitude so, therefore, become “mirror asymmetric” across the zero displacement axis.

Spatiotemporal profiles of the elastic wave were used to calculate its amplitude and group velocity.^47^^–^^51^ The measured elastic wave group velocity for all graphite phantoms was 2.18±0.2  m/s, which is in agreement with previously published OCE data.^23^

**Fig. 6:**
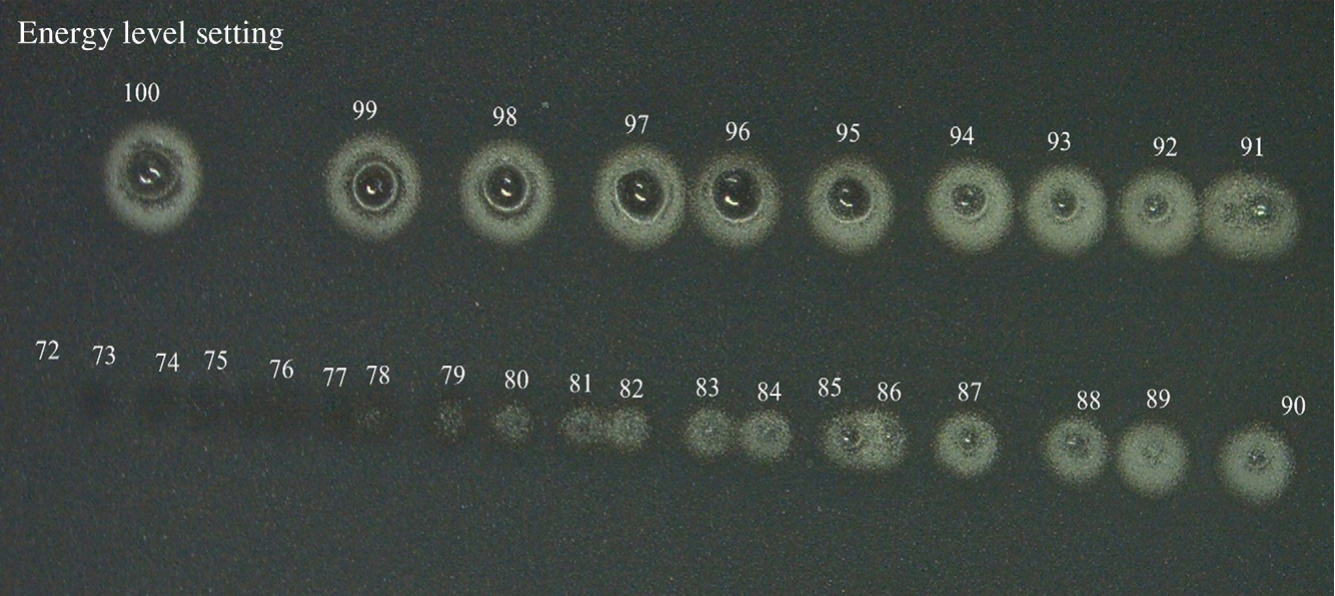
Microscopic image of the pulse laser impact on the sample surface of 1% graphite phantom (absorption coefficient is $12.21\;{\mathrm{mm}}^{-1}$). Pulse energy and fluence values for each location are indicated in Fig. 1.

The dependence of the surface wave amplitude (SWA) on laser energy was quantitatively investigated by gradually increasing the laser beam energy from 0.29 to 12.27 mJ. SWAs were averaged over three trials at each energy and on three samples of each graphite concentration. Laser pulse impact location was changed after each shot by translating the sample to ensure a fresh surface was hit with each shot. A microscope image demonstrating the impact points of the pulsed laser on the sample surface (1% graphite doping) is shown in Fig. 6. For higher pulse energies, there is clear evidence of ablation as indicated by damaged sites on the sample surface.

As the laser pulse energy is reduced, the diameter of the ablated zone decreases and eventually, at low pulse energy, no visual damage spot is observed under the microscope. However, due to the high displacement sensitivity of the LF-LCH system, elastic wave propagation is observed even at the lower energy levels despite the lack of a visible impact spot.

**Fig. 7:**
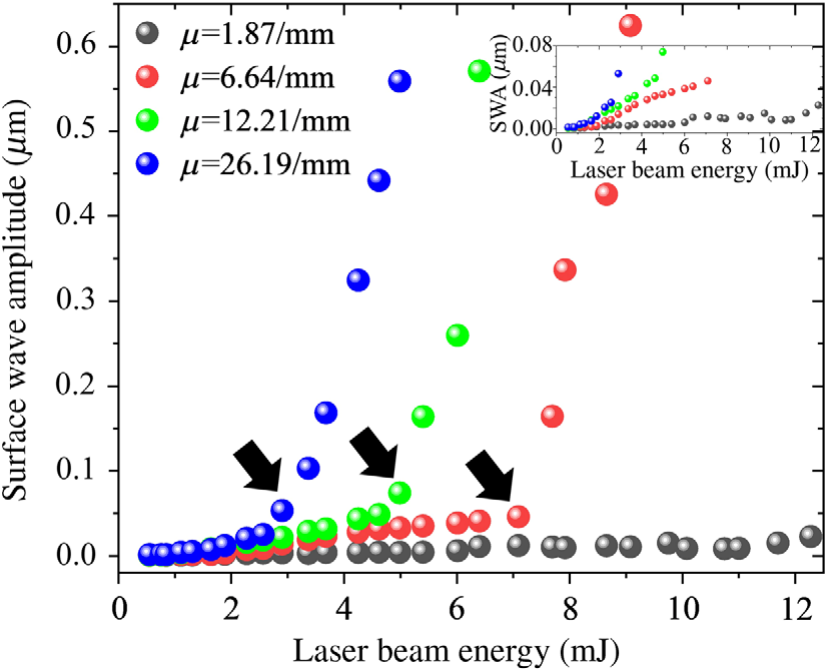
SWA for different absorption coefficients of the phantom, as a function of pulse energy. The inset shows the initial part of the SWA.

The evolution of the surface displacement as a function of laser pulse energy was used to determine the boundary between the thermoelastic and ablative regimes. Wave amplitude was calculated as the root mean square of the maximum displacements on the phantom surface caused by the laser excitation. The root mean square is calculated over the distance of 0.1 mm from the start of the imaging region (i.e., from 5 to 5.1 mm from the focus of the excitation beam). Figure 7 shows the wave amplitude versus the laser pulsed energy for different graphite phantom samples. At 0.1% graphite concentration ($\text{absorption coefficient}=1.87\;{\mathrm{mm}}^{-1}$), there is no noticeable change in temporal wave symmetry or amplitude at all laser pulse energies. As the sample absorption coefficient is increased by increasing graphite concentration, there appears a transition point energy where the slope of the wave amplitude versus pulse energy increases sharply. It has been suggested that this sharp increase in slope is due to the nonlinear nature of the ablation process.^30^ This transition point energy also corresponds closely with the energy needed to induce visible ablative damage to the sample surface, as verified via microscopy in Fig. 6. This energy level, therefore, indicates the boundary between the thermoelastic and ablative regimes at each sample absorption coefficient. The transition point energy is marked by solid arrows in Fig. 7 for each sample of the increasing absorption coefficient. Below the transition point laser energy, the mechanism of laser-induced surface wave generation is thermoelastic and above the transition point energy, it is ablative. From the results in Fig. 7, it is evident that the laser beam energy at the transition point decreases with the increased absorption coefficient of the sample. In other words, the threshold energy for crossing over from the thermoelastic regime into ablative decreases with increasingly absorptive samples and tissues.

For the proof-of-concept, the same experiments were performed with real tissue (chicken liver). Due to the curvature and roughness of the chicken liver sample, a smaller field of view with finer pixel resolution was selected for precise measurements. Additionally, as the attenuation of the elastic wave in chicken liver is high compared to agar phantoms, offset distance between the pulsed laser and the line focus was reduced from ∼5 to ∼0.5  mm, while the length of the measurement interval was decreased from 1.35 to 0.25 mm, which corresponds to the spatial resolution of 2.6  μm.

**Fig. 8:**
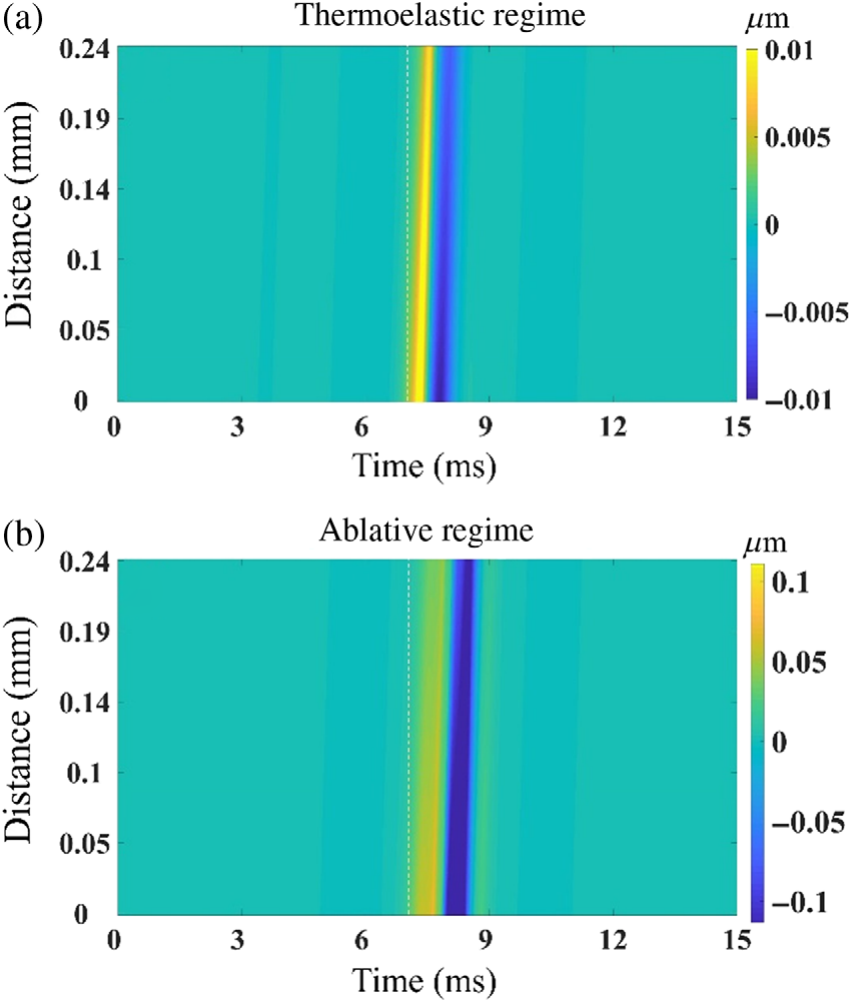
Typical spatiotemporal displacement profiles for (a) thermoelastic regime (7.92 mJ) and (b) ablative regime (11 mJ) in chicken liver.

The measured surface wave group velocity in the chicken liver is 1.48±0.16  m/s, which is in good agreement with the results of our previous study.^52^ For validation of experimental results, the experiment was repeated for an N=16 times at different positions. Full spatiotemporal displacement profiles of the laser-induced elastic wave in thermoelastic and ablative regimes in the chicken liver are shown in Figs. 8(a) and 8(b), respectively.

**Fig. 9:**
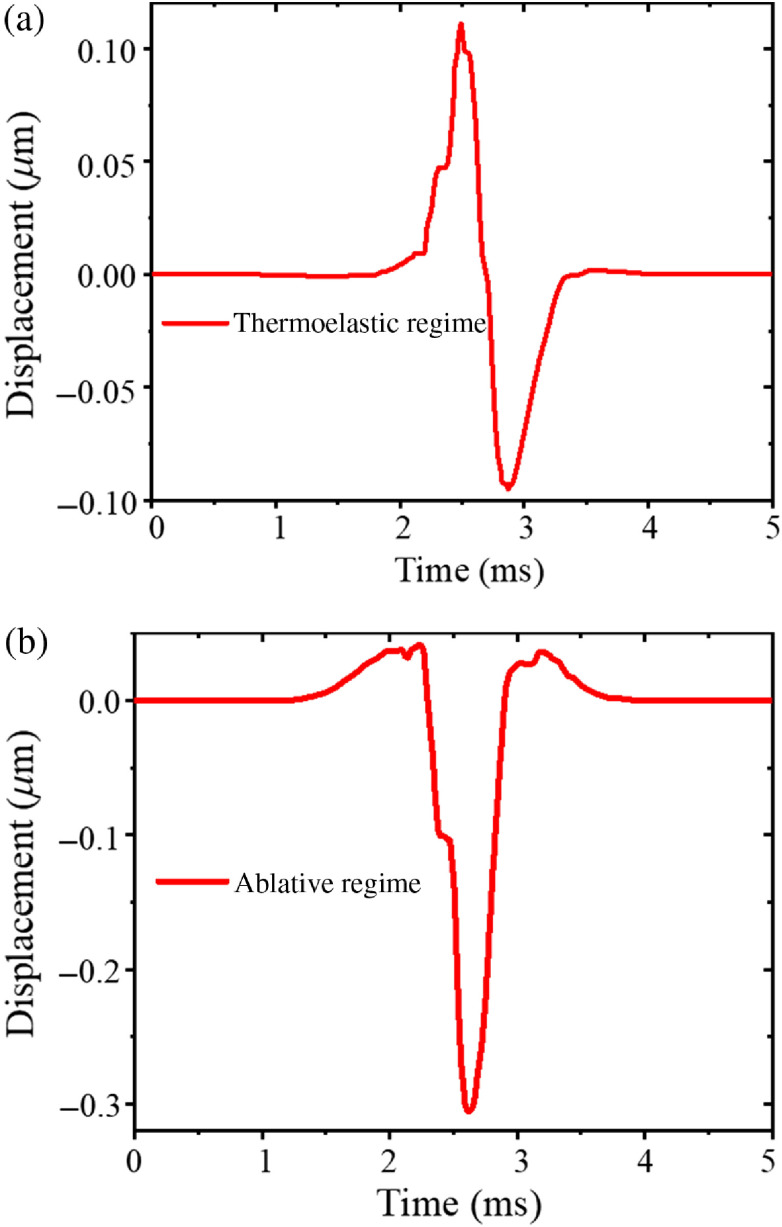
Typical temporal displacement profiles for (a) thermoelastic regime (7.92 mJ) and (b) ablative regime (11 mJ) in chicken liver.

Temporal displacement profiles of the elastic wave in the chicken liver were extracted from the spatiotemporal maps and are shown in Fig. 9. Both thermoelastic and ablative regimes share many similarities with the wave profiles in the phantom model. The temporal profiles of displacements in chicken liver appear mirror symmetric and mirror asymmetric for thermoelastic and ablative regimes, respectively.

**Fig. 10:**
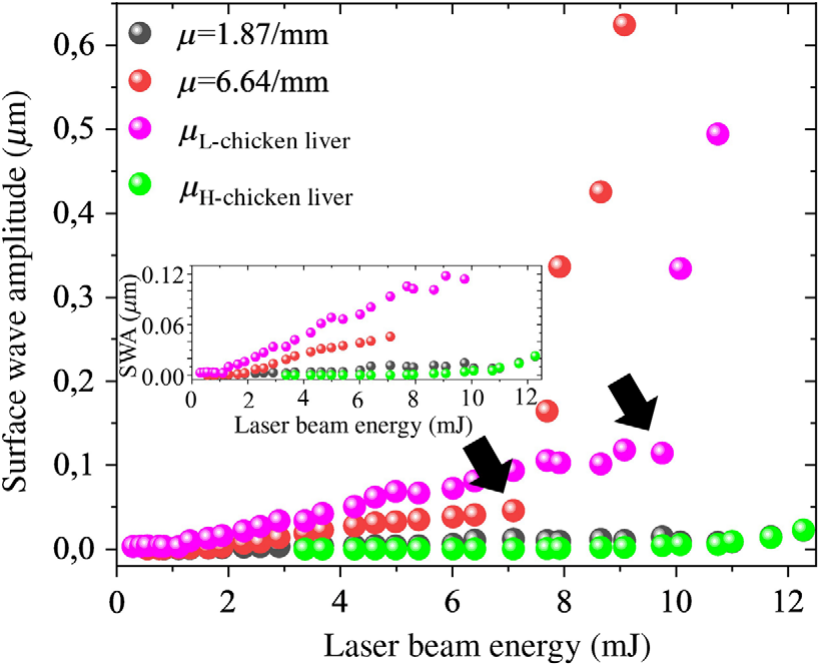
SWA comparison between chicken liver and different phantoms for two different energies of laser pulse. Initial part of the SWA is shown in inset.

As a further analysis, the SWA versus laser pulse energy in the chicken liver is compared with two graphite phantoms of absorption coefficients 1.87 and $6.64\;{\mathrm{mm}}^{-1}$, as shown in Fig. 10. Two representative data sets for chicken liver (green and magenta circles) were taken from the same sample but at different measurement locations, and two different amplitude versus laser pulse energy response plots were obtained.

One set of results shows the transition point from thermoelastic to the ablative regime, just as in the agar phantom control experiments; while the other set demonstrates no transition point energy. This result appears to demonstrate that the absorption coefficient of the chicken liver is not homogeneous across the sample surface. Comparing with the results from graphite phantoms, it appears that some locations on the liver sample exhibit an absorption coefficient of nearly $6.6\;{\mathrm{mm}}^{-1}$, while other locations likely fall below $1.8\;{\mathrm{mm}}^{-1}$.

**Fig. 11:**
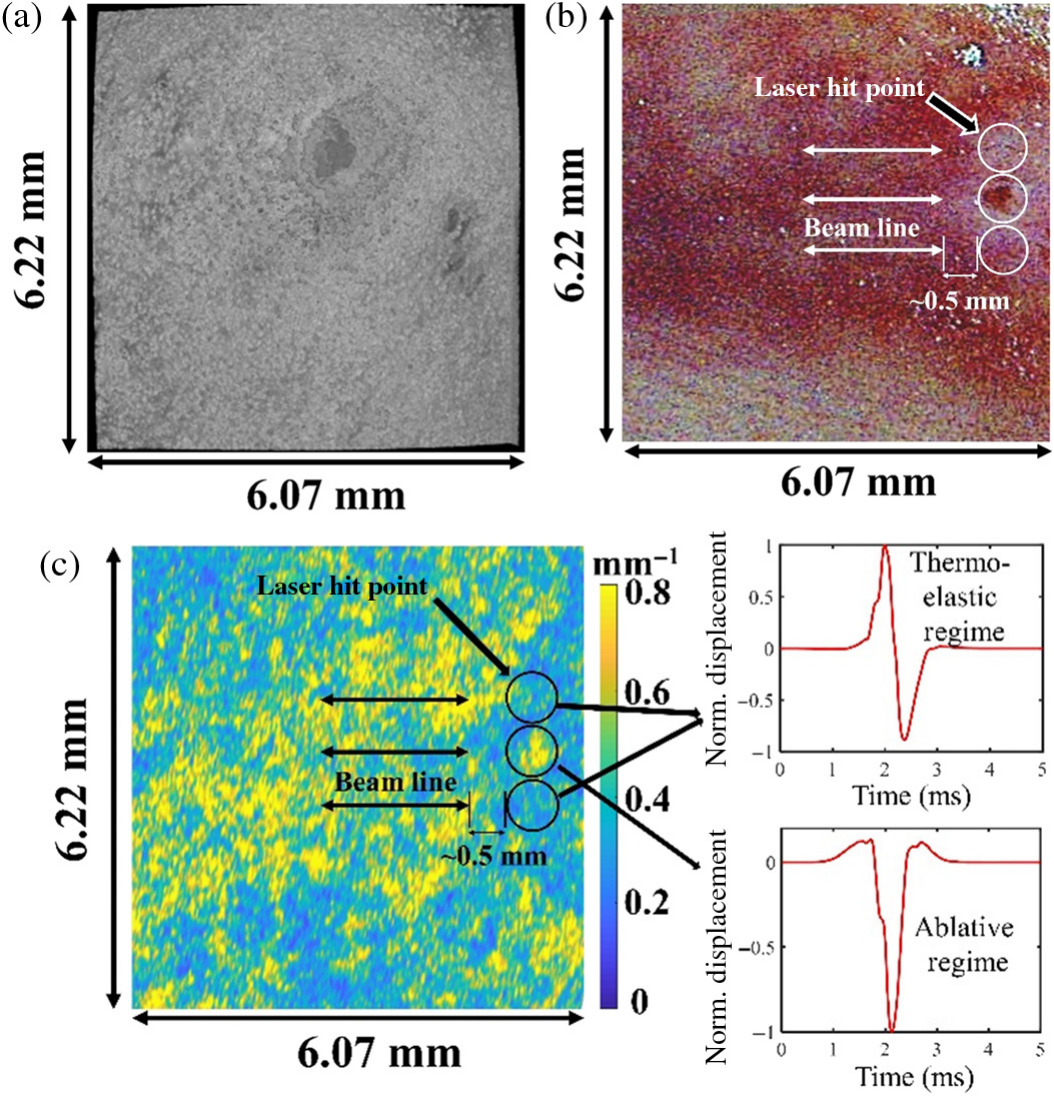
(a) Top view of the structural image of the chicken liver taken using a swept-source OCT system with central wavelength of 1310 nm. (b) Color microscopic image of the chicken liver sample with different positions of excitation and measurement. (c) Attenuation map of the chicken liver sample with excitation positions for two different sets of results indicated along with the corresponding temporal profiles.

As hemoglobin is considered the primary chromophore in the liver tissue, then it may be expected to see the absorption coefficient as high as $∼30\;{\mathrm{mm}}^{-1}$ in the locations with high blood concentration.^53^ Structural OCT images and microscope images of the chicken liver sample are shown in Figs. 11(a) and 11(b), respectively. The *en face* OCT image of the liver surface, taken at the central wavelength of 1310 nm, cannot clearly show heterogeneity in the absorption coefficient. In contrast, full-color microscopic images clearly indicate that some regions on the chicken liver are darker than others due to the strong absorption of visible light by blood. These color images qualitatively represent the heterogeneous absorption of the chicken liver sample.

An optical attenuation measurement at 1310 nm for each A-line in the OCT image^54^ is shown in Fig. 11(c). While optical attenuation at this wavelength is dominated by the scattering, OCT optical attenuation map of the chicken liver sample has a strong correlation with the optical image shown in Fig. 11(b). Netherveless, optical excitation in the areas of increased optical attenuation results in the generation of the ablative elastic waves. In comparison, the same energy of the optical excitation in the areas with reduced optical attenuation generates elastic waves in the thermoelastic regime.

## 4 Discussion

In this study, we demonstrated that the transition from the thermoelastic to the ablative regime is accompanied by the nonlinear increase in SWA as well as the transformation of the wave shape. Such results are in agreement with the results of measurements of laser-generated acoustic waves. In the low irradiance regime, before ablation takes place, the amplitude of the laser-generated acoustic wave shows a linear increase with pulse energy.^40^^,^^45^ Also, it has been shown that there is a large enhancement in the amplitude of the compressional acoustic wave generated in the ablative regime.^39^^,^^40^^,^^42^ The momentum transfer during the ablation process can be represented by a force acting normal to the tissue surface. The direction of this force on the sample surface is opposite to the force direction in the thermoelastic regime.^34^

Theoretical modeling for laser-generated ultrasound in metals has demonstrated that the resulting force on the sample surface is a combination of a time-varying normal force and the forces generated by thermal expansion.^39^^–^^45^ Therefore, the transformation of the surface wave shape shown in Figs. 5 and 9 reflects the increasing influence of the normal forces generated by the ablation of material.

Although the amplitude of the elastic waves depends on the distance between the excitation and measurement locations, we assume the estimated transition point of the wave shape is independent of this distance. Indeed, the displacement amplitude on the sample is less than a micron. Therefore, it can be considered that the deformation is in the linear elastic region, and the dependence of the amplitude on the laser energy is defined only by the absorption coefficient of the sample. However, the shape of the elastic wave presented in Figs. 5 and 9 could be different at different distances from the excitation location, taking into account the source effect and wave dispersion in the sample.

All-optical OCE has advantages of measuring tissue biomechanical properties using a single shot excitation by a laser. However, there is a valid concern about the damage of tissue at the excitation area. Since the phenomenon is basically the sudden thermal expansion of the localized tissue by absorbing the laser irradiation, the temperature at the excitation position rises sharply. Due to the laser-induced heating of the tissue, a change in microstructure in collagenous tissue has been reported.^13^^,^^14^ While moderate tissue temperature rise (below 50°C) is unlikely to induce permanent tissue damage,^55^ full characterization of tissue absorption at different wavelengths and the quantitative evaluation of maximum permissible exposure will be required before transition for *in vivo* application.

## 5 Conclusion

This study described a methodology for determining the boundary region between thermoelastic and ablative regimes of elastic wave generation. These can be used for the development of a safe method for completely noncontact, all-optical microscale assessment of tissue biomechanics using laser-induced elastic waves. With the proper selection of laser wavelengths to match the optical absorption of the target tissue, it may be possible to perform laser-induced elastography without optical damage. These results open up the potential for nondestructive all-optical OCE measurements *in vivo*.
